# Modelling Size Structured Food Webs Using a Modified Niche Model with Two Predator Traits

**DOI:** 10.1371/journal.pone.0099355

**Published:** 2014-08-13

**Authors:** Jan Klecka

**Affiliations:** 1 Departmemt of Fish Ecology and Evolution, Eawag: Swiss Federal Institute of Aquatic Science and Technology, Kastanienbaum, Switzerland; 2 Laboratory of Theoretical Ecology, Institute of Entomology, Biology Centre of the Academy of Sciences of the Czech Republic, České Budějovice, Czech Republic; 3 Department of Ecosystems Biology, Faculty of Science, University of South Bohemia, České Budějovice, Czech Republic; Northwestern University, United States of America

## Abstract

The structure of food webs is frequently described using phenomenological stochastic models. A prominent example, the niche model, was found to produce artificial food webs resembling real food webs according to a range of summary statistics. However, the size structure of food webs generated by the niche model and real food webs has not yet been rigorously compared. To fill this void, I use a body mass based version of the niche model and compare prey-predator body mass allometry and predator-prey body mass ratios predicted by the model to empirical data. The results show that the model predicts weaker size structure than observed in many real food webs. I introduce a modified version of the niche model which allows to control the strength of size-dependence of predator-prey links. In this model, optimal prey body mass depends allometrically on predator body mass and on a second trait, such as foraging mode. These empirically motivated extensions of the model allow to represent size structure of real food webs realistically and can be used to generate artificial food webs varying in several aspects of size structure in a controlled way. Hence, by explicitly including the role of species traits, this model provides new opportunities for simulating the consequences of size structure for food web dynamics and stability.

## Introduction

Animal communities form complex networks of interspecific interactions; the most traditionally studied type of such networks is the food web [1]. It is widely recognized that stability of food webs is driven by non-random features of food web structure [2]–[4]. Trophic links typically depend on the size of predators and prey [5]–[12] and it has been shown that predator-prey body mass ratios can affect food web stability [13], [14]. Hence, we need to develop models that faithfully represent the empirically observed dependence of predation links on body masses of predators and prey to gain better insights into the structure and stability of food webs.

The lack of mechanistic models using body mass to explain or predict food web structure [15], [16] is surprising given that the role of body mass for structuring food webs has been recognized already at least by Elton almost a century ago [1]. On the other hand, a family of phenomenological stochastic food web models has been developed with the intention to mimic the structure of real food webs using a set of simple rules which do not invoke any specific mechanisms. A prominent example is the niche model [17] which assumes that predators feed on a contiguous range of prey sorted along a single niche axis. The assumption of intervality has been relaxed in recent extensions of the model [18]–[20]; however empirical food webs are close to intervality [18], [21], [22], so the assumption of intervality is a reasonable simplification. It has been speculated that body mass is the key trait defining the position of species on the niche axis [7], [17] and a body mass based version of the niche model, which makes this assumption explicit was published recently [20], [23]. The original niche model and its modifications have been found to describe the structure of real food webs faithfully [17], [18], [20], [23], [24]. However, this conclusion was based almost exclusively on comparison of several summary statistics of food web structure between model generated and real food webs. The relationship between predator and prey body mass was not among them because the original niche model and most of its modifications [17], [18] do not include any information on species body masses (but see [20], [23]). However, it is widely recognized that predator and prey masses are strongly correlated in real food webs [8]–[11]. Testing whether this correlation is reproduced by theoretical food web models is of utmost importance.

There is also mounting evidence that other traits than body mass can modify the probability or strength of predator-prey links [25], such as foraging mode of the predator [26], [27]. Models relating multiple species traits to the probability of feeding interactions [25] and interaction strength [28] have already been developed. These statistical models can be directly fitted to empirical data and used to test the importance of different species traits for feeding interactions [27]. However, the focus of this paper is on stochastic models, such as the niche model and its extensions. These models have usually been compared to empirical data indirectly by various metrics of food webs structure [17], [29], although methods allowing to calculate model likelihood for directly comparing predicted and observed links have been recently developed [19], [20], [23]. These models are also prominently used in simulation studies of food web dynamics. They can be used to generate artificial food webs with specified properties; these model food webs can be used to explore the consequences of food web structure for dynamics, for example to test the effects of predator-prey mass ratios [13], [14] or connectance [30], [31] on food web stability.

Here I test whether a body mass based modification of the niche model represents size structure of feeding interactions in food webs realistically. Moreover, I introduce a novel extension of the niche model which directly includes empirically observed prey-predator body mass allometry [8]–[11]. Finally, I include a second trait modifying prey size selectivity of predators [26], [27]. The aim of these empirically motivated modifications of the niche model is to develop a simple stochastic food web model capturing the size structure of a wide range of food webs. Such a model will be valuable for exploring the consequences of size structure for food web dynamics and stability in future simulation studies.

## Materials and Methods

### The original niche model

The niche model [17], referred to as “the original niche model” here, predicts the occurrence of feeding links in a food web with 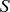 species. Each potential link is an element 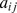 of an 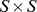 adjacency matrix 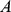, where 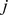 is a potential consumer of species 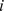. A link 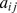 is either present (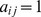) or absent (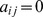). Key characteristics of the original niche model are that species can be sorted along a single niche axis, which defines a feeding hierarchy, and that the diets of consumers contain species distributed along a contiguous section of the niche axis [19]. Using only two parameters, species richness (*S*) and connectance (*C*), the original niche model generates a food web matrix using a simple set of instructions. Each species is assigned a position on a one-dimensional niche axis by drawing a random number 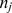 from a uniform distribution on an interval 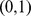, diet breadth 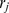 and the centre of the feeding niche 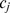 from the interval 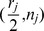. Then, feeding links are assigned to all predator-prey combinations where a prey 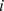 has 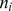 within the range of 
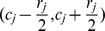
 for predator 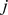. The original niche model produces interval food webs, i.e. webs where each predator feeds on a contiguous range of prey along the niche axis. Another characteristic is that constraints on possible prey a predator can eat are weak and consequently there is only a small set of forbidden links (sensu [32], [33]). A predator 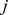 can potentially feed on any prey 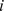 with 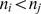 and also on many prey with 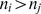; up to 50% of a predator's diet can have higher 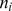 than the predator's 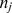.

Although it has been shown that body mass can be used as a basis for sorting species in food webs [5]–[7], [20], [23], [24], the original niche model does not assume that predator-prey interactions depend on body mass. The niche value is an abstract value, which limits the applicability of the niche model to address specific biologically relevant questions about the role of body mass for food web stability. To circumvent this problem, it has been proposed that one can transform the niche value to body mass of species 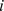 as 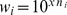, where 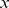 is a parameter fixing maximal predator-prey mass ratio, which is 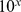 [14]. However, a direct way to incorporate body mass into the niche model was recently developed [20], [23] (see below).

### The body mass based niche model

A body mass based modification of the niche model uses body mass to calculate species niche values [20], [23]. Several body mass based versions of the niche model developed by Williams et al. [20] also include other changes to the niche model. However, in its simplest form used here, the only difference compared to the original niche model is that the niche value is a function of body mass:
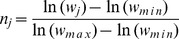
(eq. 1)where 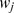 is body mass of species 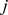, 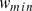 and 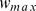 is the mass of the smallest and the largest species in the food web, respectively. I will refer to this model as the “body mass based niche model”. Stouffer et al. [18], Allesina et al. [19] and Williams et al [20] introduced further modifications of the niche model allowing for non-contiguous diets but I do not consider this aspect here because diets in real food webs have usually high degree of intervality [18], [21], [22]. Hence, the body mass based niche model explicitly assumes that body mass controls trophic position of individual species but otherwise the structure of the food webs generated by this model is identical to the original niche model. Code to generate food web structure (written in R) is avalaible as Supporting Information (Script S1).

### Introducing the two traits allometric model

**Figure 1:**
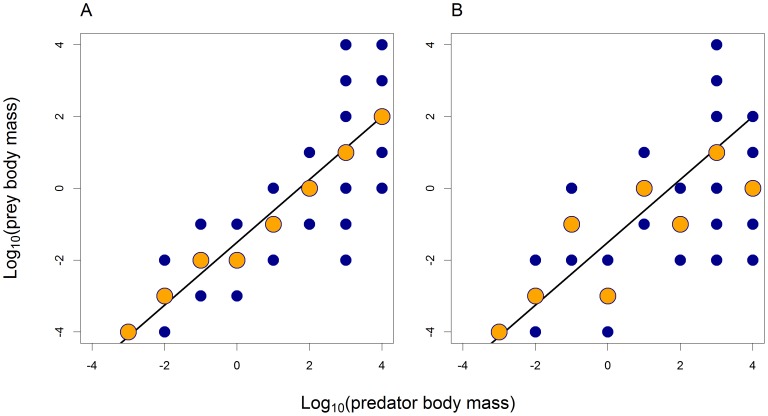
Comparison of two food webs with different importance of body mass for predator-prey interactions. An illustrative example of a small food web (9 species); each species is displayed on the x-axis as a predator and on the y-axis as a prey (the smallest species is a non-predatory basal species). The black line shows an allometric relationship between predator mass and optimal prey mass, large orange circles show the prey closest to the optimal prey mass for individual predators and smaller blue circles show other predator-prey links present in the network. In (A) optimal prey mass depends allometrically only on predator mass. In (B) optimal prey mass depends also on a second trait of a predator (a foraging trait), which causes deviations from the prey-predator body mass allometry depicted by the black line. Many real food webs are characterized by such a blurred relationship between prey and predator body masses.



Here I propose further extensions of the body mass based niche model to incorporate predator-prey body mass allometry well known from empirical data and include a second predator trait to move from a simple body mass dependence of predator-prey interactions to multiple-trait based approach [25], [27], [28]. The centre of the feeding range 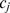 of a predator 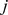 is constrained to be a function of predator's body mass 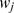. Empirical data show that predator's optimal prey mass 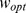 is allometrically related to its body mass 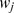:
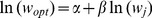
(eq. 2)for a predator 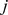 [8]–[11]. This empirically derived predator-prey mass allometry is explicitly included in the model: the centre of the predator's feeding range 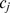 equals to the niche value 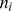 of a prey 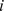 with mass 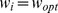; i.e., predator's feeding range 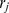 is centred on a prey with the optimal mass for a given predator:
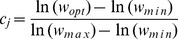
(eq. 3)where 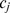 is the centre of predator's feeding niche and 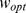 is the optimal prey mass for predator 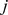. The second modification I propose is an inclusion of a second predator trait 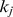 which modifies the optimal prey mass 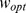 for a predator 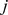. It models a situation when equally sized predators differing in foraging mode or another trait prefer prey of different sizes [26], [27]. An illustrative example is shown in Figure 1. Specifically, I modify the allometry of predator mass and optimal prey mass (eq. 2):

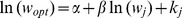
(eq. 4)

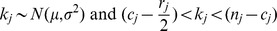
where 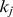 denotes the effect of a second trait on the optimal prey mass for a predator 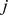. I will refer to this model as the “two traits allometric model”. I treat 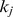 as an unknown, or latent, trait [25] in the simulations. Because it is related to foraging behaviour of predators, I call it here a “foraging trait”. I include the effect of the foraging trait 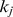 on optimal prey mass 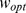 of each predator by drawing a random value from a truncated normal distribution and inserting it into the formula above (eq. 4). I used mean 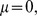 various values of standard deviation 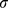; lower bound and upper bound was set to ensure that predator's 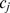, which is a function of 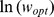 (see eq. 3), lies within the range set by the basic constraints of the niche model; i.e. in the interval 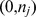. The effect of the second trait is independent of predator body mass (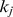 and 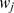 are uncorrelated). The strength of the effect of the second trait 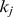 relative to body mass 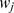 can be manipulated by the choice of 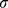 of the truncated normal distribution used to generate the values of 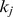. Thanks to this feature, the two traits model covers the whole range of possibilities from the strictly size-dependent predation when 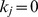 (eq. 2) to the very weak size constraints of the body mass based niche model (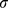 very large). For sufficiently large values of 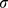 the two traits model converges to the body mass based niche model described above [20], [23], where the centre of the feeding niche 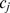 is drawn uniformly from the interval 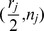. Intermediate cases can be easily achieved by an appropriate choice of the standard deviation 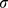. A function rtruncnorm in the truncnorm package for R was used to sample from the truncated normal distribution [34]. Code to generate food web structure (written in R) is avalaible in Script S1.

### Analysis of the structure of model food webs

I carried out extensive simulations in R 2.15.1 [35] to analyse the structure of model food webs generated by the body mass based niche model and its modifications described above. I also compared the results to those based on the original niche model where body mass is calculated post hoc from niche values as 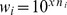 for species 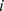, where 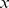 is a parameter fixing maximal predator-prey mass ratio, as described by Heckmann et al. [14]. To generate food webs with predator-prey interactions depending on species body mass using the body mass based niche and two traits models, I first set the number of species (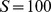 in the main results) and their body masses. Since the distribution of body masses is known to follow approximately a log-normal distribution, I assigned body masses to all species by randomly generating numbers from a log-normal distribution with 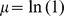 and 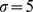; the value of 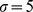 corresponds to total size range in the food web on average 10 orders of magnitude, which may represent, for example, a freshwater food web with species ranging from small zooplankton (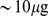) to large fish (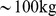). The effect of total mass range on the resulting food web structure was further explored by changing the value of 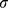 of the log-normal distribution of body masses. In all results reported here, I set connectance 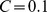 (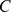 effectively controls the mean diet breadth of consumers) and excluded food webs with 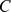 differing from the target value by more than 3%, which is a standard procedure (e.g. [17], [31]). In the case of the two traits allometric model, I also fixed the values of parameters describing the allometry of predator and prey masses (eq. 4). I set 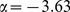 to get a predator-prey mass ratio of 37.7 for a predator of body mass 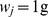 and 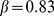; these values are based on published analyses of a large database of predator-prey interactions [8]–[11]. I set the standard deviation 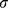 of 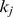 to several different values, which leads to a range of situations differing in the strength of the size dependence of feeding links. I also included the special case of 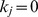, where the effect of the second trait is absent and optimal prey mass depends only on predator mass (eq. 2).

In the case of the original niche model, a different approach had to be used. I set the number of species and connectance as above and generated food web structure. Then, I calculated body masses form the niche values (instead of calculating niche values from body masses) using a method proposed by Heckmann et al. [14]. Here, 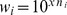 for species 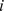, where 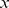 is a parameter fixing maximal predator-prey mass ratio, which is 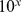. I set the value of 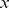 to match the mean value of total body mass range in the other models. This leads to a close correspondence between the original and body mass based niche models. Comparison of the results between these two models thus shows the consequences of the choice to (1) either generate niche values first and then use them to calculate body masses or (2) to use the opposite, arguably more natural, procedure of specifying species body masses first and then using them to calculate niche values. Distribution of body masses differs in these two cases because in the original niche model I calculated body masses as 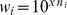 [14] and 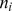 is uniformly distributed on the interval 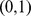. Hence, 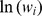 is uniformly distributed, while in the body mass based niche and two trait models, body masses were generated from a log-normal distribution.

Food webs generated by the models were compared using several measures of network structure. I analysed the prey-predator body mass allometry using standardized major axis regression (SMA regression), which is frequently used to estimate allometries because it treats errors in both the explanatory and response variable equally [36]; the package smatr for R was used [37]. The analysis of size structure of model food webs was complemented by a comparison of twelve summary statistics commonly used to describe food web topology (e.g. [16], [17], [29]). The metrics used were: the proportion of basal, intermediate and top species, the proportion of herbivores, mean and maximum trophic level, mean omnivory level, clustering coefficient, standard deviation of generalism and vulnerability, maximum diet similarity and mean path length (the definitions follow [16]). I refrained from comparing these metrics directly to real data because improving the, already good, fit of the niche model was not an objective of this paper and because the utility of such comparisons for evaluating food web models is limited [38]. I instead focused on comparing the different models among themselves to test whether the changes in size structure in these models lead to changes in other structural properties.

Species richness and connectance were kept constant, although these parameters are important for addressing other questions, such as stability and robustness of food webs [13], [39]. In the simulations reported here, species richness had no effect on the results and connectance only controlled mean diet breadth of predators, which slightly changed the quantitative output of the simulations but had no effect on comparing the models. Values of 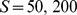 and 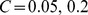 were tested in additional simulations (results not shown) to verify that the results and conclusions presented here are robust.

## Results

### Body mass structure of model food webs

**Figure 2:**
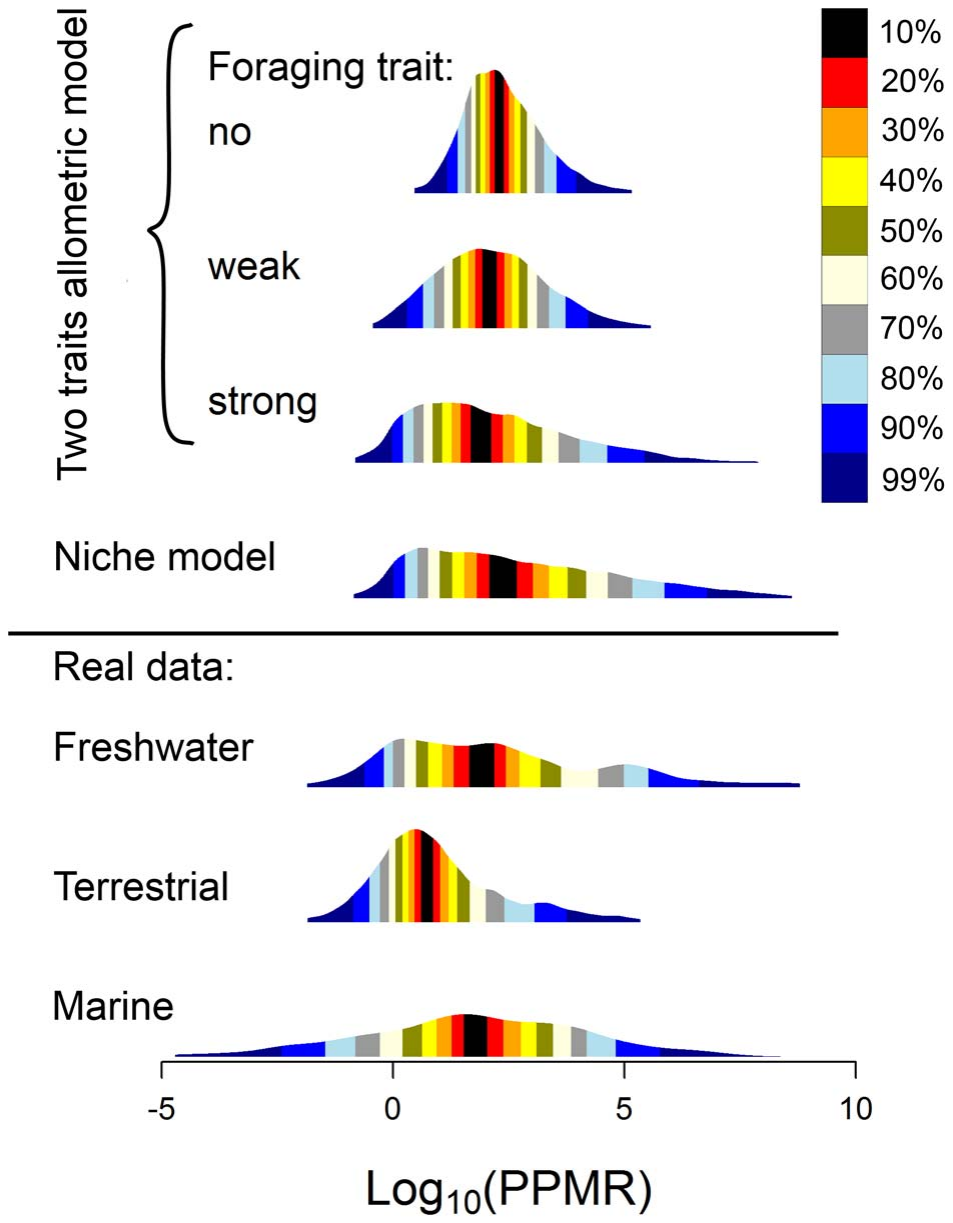
Predator-prey body mass ratios in model food webs and real data. The distributions of predator-prey body mass ratios produced by different models and real data from freshwater, marine and terrestrial predatory interactions (data from [8]). In the case of model data, results of 200 simulations of food web matrices with 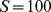 species and 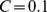 are presented for each model. In the case of the two traits allometric model, body masses were drawn from a lognormal distribution (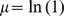 and 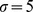); this setting leads to the total body mass range in individual food webs ca. 10 orders of magnitude). Optimal prey mass is given as 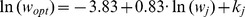, where 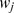 is predator mass and 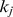 denotes the effect of a predator's foraging trait on the optimal prey mass. Settings of the foraging trait are 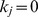 (no effect), 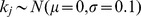 and 
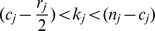
 (weak effect), and 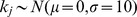 and 
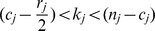
 (strong effect). See the methods section for more details. Plot shows estimated probability density functions with colours representing intervals with a given percentage of observations around the median (see the legend).

Both the original niche model and the two traits model produce food webs with the distribution of predator-prey body mass ratios (PPMR) comparable to empirical data from different habitats (Figure 2). Food webs generated by the original niche model have median PPMR = 253.8, while food webs produced by the two traits allometric model with no, weak and strong effect of the foraging trait have median PPMR = 191.4, 122.4 and 79.1, respectively. The empirical data have median PPMR = 87.1, 5.3 and 62.6 for freshwater, terrestrial and marine data, respectively. With increasing importance of the role of the foraging trait, the constraints set by the allometric dependence of optimal prey mass on predator mass are getting weaker, which leads to broader range of PPMR values (Figure 2). A notable difference from the empirical data is that all the models predict smaller numbers of interactions where predator is smaller than its prey than observed in empirical food webs (negative values of 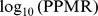 in Figure 2).

**Figure 3:**
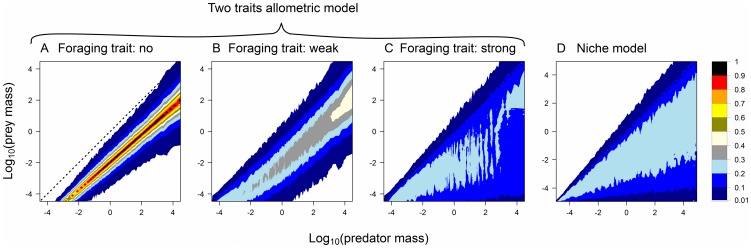
The two traits allometric model produces food webs with varying size structure. Results of 1000 simulations of food web matrices with 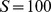 species and 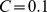 using the same models as in Figure 2. The dotted diagonal line denotes situations when prey is of the same size as its predator; values above this line mean that predator is smaller than its prey. The colours show the probability of occurrence of feeding interactions between predator and prey of a given size in the simulated food webs (see the legend). The difference of values in (C) and (D) is small; for 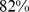 of corresponding predator-prey pairs the difference in link probability is less than 0.05.

Individual models differ in the strength of the effects of predator and prey mass on the probability of occurrence of individual feeding links; also the set of “forbidden” links (i.e. links that never occur) varies (Figure 3). In the case of the two traits allometrical model with no effect of predator foraging trait, all interactions are constrained to a narrow band around the allometry of optimal prey and predator mass, and probability of the occurrence of feeding links decreases sharply with increasing difference from the allometry (Figure 3 A). With increasing importance of the predator foraging trait (and hence decreasing role of body mass constraints), the set of forbidden links decreases (Figure 3 B and C). In the original niche model, predators can feed on a wide range of prey and consequently, all links have a low probability of occurrence (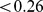) (Figure 3 D), as in the two traits allometric model with strong effect of predator foraging trait (Figure 3 C). The dependence of link probability on predator and prey masses holds independently on the total body mass range of species in a food web (not shown).

The prey-predator body mass allometry in model food webs with total body mass range of 10 orders of magnitude (Figure 2 and Figure 3) was similar in different models. The intercept estimated by SMA regression was −2.1, −1.9, and −2.1 in the two traits allometric model with no, weak and strong effect of predator foraging trait and −2.6 in the original niche model. The slope of the allometry was 0.91, 0.92, and 0.94 in the two traits allometric model with no, weak and strong effect of predator foraging trait and 0.98 in the original niche model. However, as already clear by the inspection of Figure 3, the amount of variance of prey body masses explained by predator body mass varied considerably: 82.7%, 58.8%, and 26.5% in the two traits allometric model with no, weak and strong effect of predator foraging trait and 35.1% in the original niche model.

**Figure 4:**
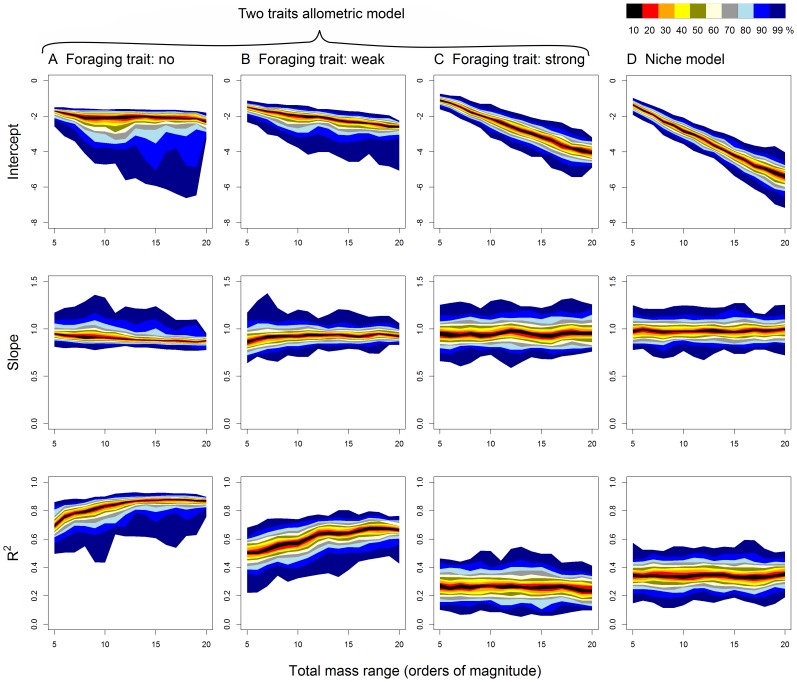
The dependence of the parameters of prey-predator body mass allometry on the total mass range of the food webs. The intercept, slope and explained variance of the dependence of 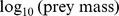 on 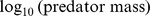 are plotted against the total mass range of the food webs. Parameters were estimated by standardized major axis regression. The same set of model food webs as in Figure 2 was used. Colours represent intervals with a given percentage of observations around the median (see the legend).

Total body mass range of the food webs (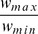) affected the estimated intercept of the prey-predator body mass allometry in the original niche model (Figure 4 D). There was a sharp decrease in the intercept with increasing size range of the food webs; the same holds for the two traits allometric model with strong effect of the foraging trait (Figure 4 C). On the other hand, the intercept was almost independent of the total mass range of the food webs generated by the two traits allometric model with no or weak effect of the foraging trait (Figure 4 A and B). The slope of the prey-predator body mass allometry and the amount of explained variance were not or only weakly dependent on the total size range of the food webs (Figure 4).

**Figure 5:**
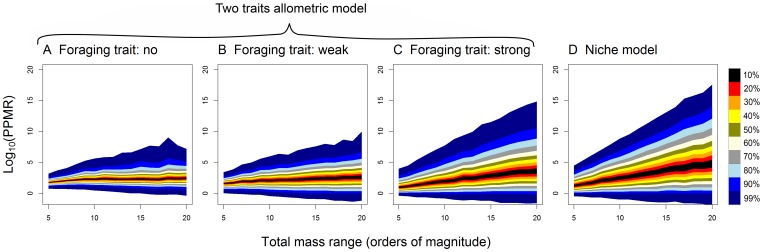
Predator-prey body mass ratios depend on the total mass range of the food webs. Plots show the values of predator-prey body mass ratios plotted against the total mass range of the food webs (
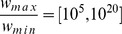
). The total mass range was manipulated by changing the value of 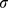 of the log-normal distribution of body masses in the case of the two traits allometric model and by changing the values of the parameter 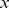 in the case of the niche model (see methods for details). Colours represent intervals with a given percentage of observations around the median (see the legend).

Mean PPMR and the range of PPMR values increased considerably when the total body mass range was increased from 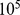 to 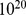 in the original niche model and the two traits allometric model with strong effect of the foraging trait (Figure 5 C and D). On the other hand, mean PPMR was independent of the total mass range and the range of PPMR values changed less in the two traits allometric model with no or weak effect of the foraging trait (Figure 5 A and B).

### Summary statistics of food web structure

**Figure 6:**
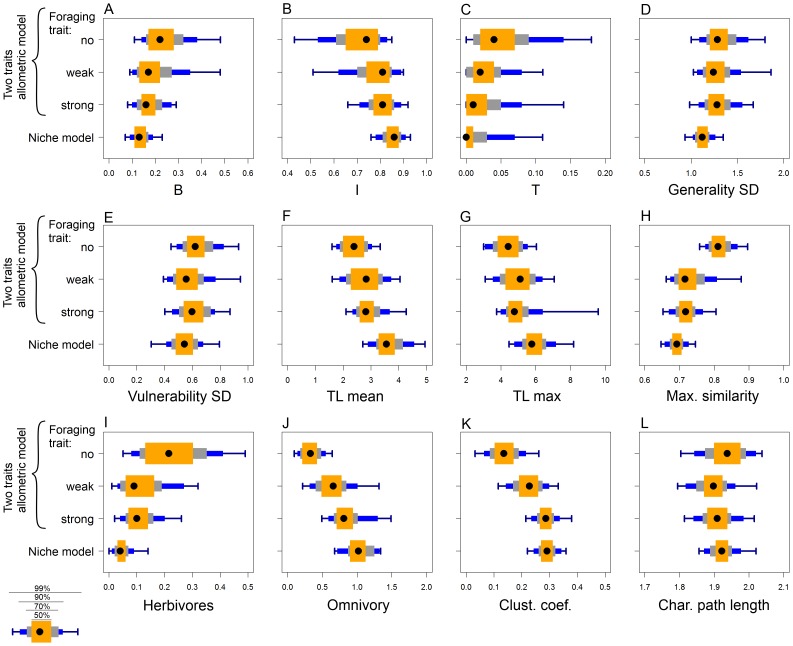
Comparison of metrics of food web structure among different models. Box-plots show the values of twelve metrics of food web structure calculated for a set of 200 food webs generated by each of the three settings of the two traits allometric model and the niche model with 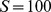 species and 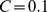. The same set of model food webs as in Figure 2 was used. The metrics shown are: proportion of basal (A), intermediate (B) and top (C) species, standard deviation of generality (D) and vulnerability (E), mean (F) and maximum (G) trophic level, maximum diet similarity (H), proportion of herbivores - i.e. consumers of basal species (I), mean omnivory level (J), clustering coefficient (K) and mean characteristic path length (L). Box-plot shows estimated intervals around the median (black circle) where a given percentage of observations lies: see the legend in the lower left corner.

Most summary statistics of food web structure varied systematically from the two traits allometric model with no effect of the foraging trait to the model with strong effect of the foraging trait (Figure 6). However, the distributions of all summary statistics overlapped (99% intervals overlapped in all metrics; in most cases the overlap was much larger), so the difference between model settings was not significant. The largest difference between the model with no and strong effect of the foraging trait was found in the case of maximum similarity (Figure 6 H), which reflects the fact that similarly sized predators have very similar diets when predator-prey links are driven only by body mass. The comparison of the two traits allometric model with strong effect of the foraging trait and the original niche model revealed no significant differences (Figure 6). However, it is noteworthy that the original niche model tends to produce slightly less basal, herbivorous and top species (median number of top species is zero) and more intermediate species (Figure 6 A, B, C and I). This pattern is connected to slightly higher level of omnivory in the original niche model food webs (Figure 6 J).

**Figure 7:**
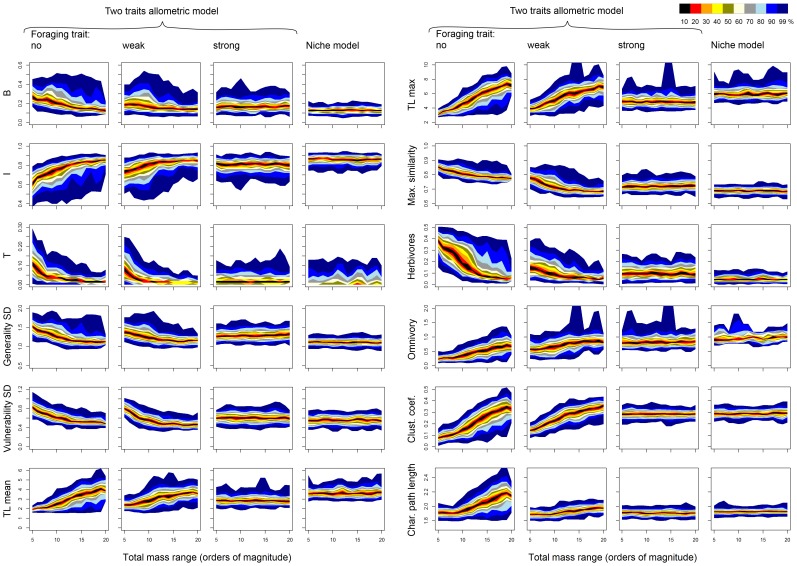
The dependence of metrics of food web structure on total body mass range in the food webs. The values of the same 12 metrics as in Figure 6 are plotted against the total body mass range in the food web (orders of magnitude) for individual models. Colours represent intervals with a given percentage of observations around the median (see the legend in the upper right corner).

**Figure 8:**
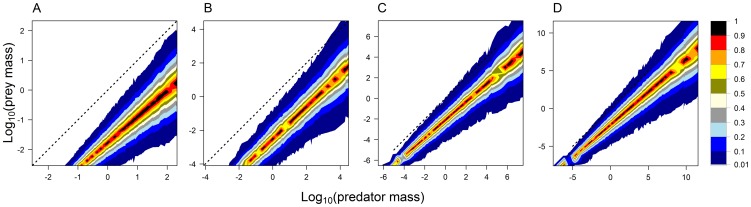
The dependence of size structure of the two traits allometric model with zero effect of the foraging trait on total body mass range in the food web. Results of 1000 simulations of food web matrices with 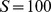 species and 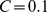 for four different values of the total body mass range: 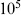 (A), 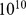 (B), 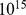 (C), and 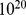 (D). The dotted line denotes situations when prey is of the same size as its predator; values above this line mean that predator is smaller than its prey. The colours show the probability of occurrence of feeding interactions between predator and prey of a given size in the simulated food webs (see the legend).

Summary statistics of food web structure varied with the total body mass range in the case of the two traits allometric model with no or weak effect of the foraging trait, while they were independent of the total body mass range in the original niche model and in the two traits allometric model with strong effect of the foraging trait (Figure 7). The observed sensitivity of the food web metrics to changes in the total body mass range results from the fact that prey mass depends allometrically on predator mass with a slope 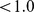 in the case of the two traits allometric model with no or weak effect of the foraging trait. As the total body mass range increases, small predators become more similar in body mass to their prey and large predators become less similar to their prey (Figure 8). This alteration of the size structure of the food webs results in changes in summary statistics of food web structure (Figure 7).

## Discussion

A plethora of different types of models seeking to explain food web structure has been developed in last several decades. Although truly mechanistic models attempting to predict food web structure based on optimal foraging have been developed recently [15], [16], most of the models published so far are stochastic phenomenological models which generate more or less random food webs using a simple set of instructions. A prominent example is the niche model [17] which produces model food webs successfully mimicking real food webs according to comparisons of summary statistics, such as the proportion of top, intermediate and basal species to real data [17], [29]. A pitfall of this approach is that a model may reproduce chosen metrics faithfully but still may fail to capture other untested aspects of food web structure; one of these is the relationship between predator and prey body mass. This paper aimed to evaluate the performance of the original niche model and its new empirically driven extensions in describing size structure of real food webs.

### How well do the models capture real-world predator-prey body mass relationships?

The most apparent difference between real data and predator-prey mass ratios (PPMRs) in model generated food webs is that real food webs seem to contain a large proportion of predators feeding on prey larger than themselves (Figure 2). This could be partly an artifact caused by aggregating data on the species level instead of analysing them on the individual level, because many species, such as fish, grow in size over many orders of magnitude during ontogeny. When adults of a small species feed on juveniles of larger species, but the food web is analysed using species average or asymptotic body masses, we may draw a link where a predator is smaller than its prey. However, at the individual level, the predator may actually be larger than its prey [40], [41]. Moving towards individual-based approaches in food webs is highly desirable for gaining novel insights [42], but most currently available data and current food web models use the species level.

The original niche model does not capture the relationship between predator and prey masses faithfully compared to empirical data. When the total mass range is set to 10 orders of magnitude, mean PPMR and the slope of the prey-predator mass allometry estimated by SMA regression correspond well to real data (Figure 2, [8]–[11]), although there is a lot of variation among individual simulations. However, the variance of prey body mass explained by predator body mass was only ca. 35% in the original niche model, compared to 86% in the analysis of empirical data by Brose et al. [8], which means that real food webs have much tighter prey-predator body mass allometry. Moreover, the intercept of the allometry and the range of PPMR values strongly depended on total range of body masses in the food web. This result stems from the fact that the niche model imposes only very weak constraints on predation – a predator can feed on any prey which is smaller than the predator and also on many larger prey species. Hence, maximum predator-prey mass ratio increases linearly with increasing total range of body masses in the food web (Figure 4). Cases when a large predator feeds on very small prey, such as whales feeding on krill are recognized as exceptions from general size-dependence of predatory interactions [8]–[12]. High sensitivity of the niche model predictions of the prey-predator body mass allometry to changes in the total mass range (Figure 5) limits the utility of the niche model to study the effect of predator and prey masses on food web stability [13], [14].

The two traits allometric model generates food webs that capture predator-prey body mass relationships in real food webs well. When the effect of predator foraging trait is absent and predation is governed exclusively by prey-predator body mass allometry, predator mass explains on average 82% of prey mass variance, which corresponds to empirical data analyses by Brose et al. [8] (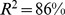). The proportion of variance in prey mass explained by predator mass decreases as the effect of the predator foraging trait increases. Importantly, the prey-predator mass allometry and mean PPMR do not depend on the total mass range unlike in the original niche model. However, with increasing total mass range, the range of PPMR values increases (Figure 5) as a natural consequence of the fact that because connectance remains fixed in the simulations (i.e. the mean number of prey species consumed by a predator does not change with body mass range), predators feed on prey of body masses deviating more strongly from the optimal prey mass. However, this effect is much weaker than the increase of the range of PPMR values in the body mass based niche model (Figure 5). This effect would be moreover counteracted if total body mass range increases in food webs with increasing species richness and decreasing connectance, which has been empirically observed [43]–[45]. By changing the value of the standard deviation of the truncated normal distribution used to assign the values of the foraging trait 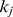, the model can cover the whole spectrum of food web size structure from that typical for the original niche model (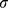 very large) to that observed when the effect of foraging trait is absent and interactions are driven exclusively by body mass (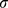 close to 0). The role of body mass also varies among real food webs from different habitats and with different types of consumers [8], [9], [11], [12], [23]. In some food webs, the role of other traits may be even more important that the role of body mass [12]. The two traits allometric model captures a large part of this natural spectrum of food web structures depending on its parameter values.

### Are there differences in food web structure among the models?

The comparison of twelve standard descriptors of food web structure (Figure 6) shows that although the three models differ in the size structure, most summary statistics of food web structure have similar values. These results suggest that many of these metrics are not tightly coupled with the size structure of food webs. This allows a model that makes not entirely realistic assumptions about the role of body mass in structuring food webs (i.e. the body mass based niche model) to provide very good fit to empirical food webs when the fit is judged by comparing values of summary statistics of food web structure [17], [29]. There are other means of comparing food web models to empirical data, such as comparing the proportion of correctly predicted links or calculating model likelihood [16], [19], [38], [46]. However, fitting models to real data on food webs remains a tricky task [38] which may suffer from limitations beyond the choice of the appropriate metrics. Sampling effects may bias empirical descriptions of food web structure [47], [48], which is certainly true also in the case of size structure because many common sampling methods are size selective and provide a distorted view of community composition in aquatic (e.g. [49]–[52]) as well as terrestrial (e.g. [53]–[55]) habitats. Moreover, I argue that stochastic structural food web models are better suited for studies simulating food web dynamics than for fitting to real data because they only describe food web structure without providing mechanistic explanations [38]. For the purpose of simulation studies, it is important that manipulating the size structure of food webs using the two traits allometric model has only minor consequences for other structural properties of food webs.

The original niche model [17] produces webs that are interval and species with similar niche value share predators but can still feed on different prey. Diet contiguity is retained in the two traits allometric model for the sake of simplicity because real food webs usually have high degree of intervality [18], [21], [22]. The two traits model allows species with similar body masses to feed on different prey because the second trait affects prey size preference of the predators. The inclusion of such a predator trait was motivated and is supported by empirical studies showing that foraging mode affects prey size selectivity of some predators. For example, filter-feeding zoopklanton prefers smaller prey than equally sized raptorial zooplankton [26] and suctorial predatory aquatic insects feed on larger prey than chewing predators of equal mass [27].

### Using these models to understand real food webs

The two traits allometric model could be used to fit empirical data by specifying masses of individual species based on measurements and by choosing the parameters of the allometry of optimal prey mass and predator mass corresponding to a real food web in question or by optimizing parameter values to obtain the best possible fit. Stochastic food web models have been fitted to empirical data in this fashion [19], [20], [23], [46], but the value of such comparisons is limited because the models are purely phenomenological and do not provide mechanistic insights [38]. It has been shown that any model obeying a few simple constraints will fit real food web data similarly well [56]. However, fitting models such as the body mass based niche model and the two traits allometric model can help to identify predators that deviate from predator-prey allometry obeyed by the rest of the species in the food web, have unusual diet breadth, etc. [20], [23]. Such explorations might pinpoint species worth studying in more detail to explain why are they different. This may be a fruitful way of connecting phenomenological food web models with real-world data to gain novel biological insights.

The explicit inclusion of species body mass to the niche model [20], [23] and the possibility to manipulate size structure of food webs by directly including prey-predator mass allometry and other modifying traits into the model, as demonstrated in this paper, makes these models an increasingly flexible tool. The importance of body mass for structuring real food webs varies [8]–[12], so understanding the dynamical consequences of size structure is a key question of food web research [13], [14]. The two traits model allows us to generate artificial food webs varying in size structure, which can be used in simulation studies of food web stability. Hence, this and similar models hold a significant promise for addressing the role of size structure of food webs for their dynamics and stability in a more detailed and direct way then was possible in previous studies.

I did not include prey traits other than body mass in the modifications of the niche model discussed in this paper for the sake of simplicity. However, recent results suggest that closely related prey are consumed by similar sets of predators but closely related predators have less similar diets; this asymmetry of the role of species similarity may be a consequence of differences in the roles of predator and prey traits [12]. New approaches in food web modelling based on multiple species traits [25], [28], [57] clearly hold a significant promise in moving towards more realistic descriptions of food web structure as they enable us to predict interaction strength based on biological traits of interacting species [27]. Different types of food web models can complement one another to address a broad range of questions using appropriate tools; such as trait-based models to predict individual feeding links and interaction strengths and stochastic structural models to study the links between structure and dynamics by simulation studies.

## Supporting Information

Script S1 **R script containing the code for the models used in the paper.** The script contains a function niche.model which generates a food web using the original niche model and calculates species body masses from the niche values, a function niche.replicated which produces a list of n webs based on the original niche model, a function two.traits.model which generates a food web using the two traits allometric model and a function two.traits.replicated which produces a list of n webs based on the two traits allometric model.
(R) Click here for additional data file.
